# Association between glucocorticoid use and all-cause mortality in critically ill patients with heart failure: A cohort study based on the MIMIC-III database

**DOI:** 10.3389/fphar.2023.1118551

**Published:** 2023-01-12

**Authors:** Jia-Liang Zhu, Liang Hong, Shi-Qi Yuan, Xiao-Mei Xu, Jian-Rui Wei, Hai-Yan Yin

**Affiliations:** ^1^ Department of Intensive Care Unit, The First Affiliated Hospital of Jinan University, Guangzhou, Guangdong, China; ^2^ Guangzhou Women and Children’s Medical Center, Guangzhou Medical University, Guangzhou, Guangdong, China; ^3^ Department of Intensive Care Unit, Nanjing First Hospital, Nanjing Medical University, Nanjing, China; ^4^ Department of Neurology, The First Affiliated Hospital of Jinan University, Guangzhou, Guangdong, China

**Keywords:** glucocorticoids, heart failure, all-cause mortality, retrospective cohort study, MIMIC-III database

## Abstract

**Background:** Heart failure (HF) is the terminal stage of various heart diseases. Conventional treatments have poor efficacy, and diuretic resistance can present. Previous studies have found that the use of glucocorticoids can enhance the diuretic effect of patients with heart failure and reduce heart failure symptoms. However, the relationship between glucocorticoid use and mortality in patients with heart failure in intensive care units is unclear.

**Objectives:** The aim of this study was to determine the association between glucocorticoid use and all-cause mortality in critically ill patients with heart failure. Methods: The information on patients with heart failure in this study was extracted from the MIMIC-III (Medical Information Mart for Intensive Care-III) database. Patients in the glucocorticoid and non-glucocorticoid groups were matched using propensity scores. The Kaplan-Meier method was used to explore the difference in survival probability between the two groups. A Cox proportional-hazards regression model was used to analyze the hazard ratios (HRs) for the two patient groups. Subgroup analyses were performed with prespecified stratification variables to demonstrate the robustness of the results.

**Results:** The study included 9,482 patients: 2,099 in the glucocorticoid group and 7,383 in the non-glucocorticoid group. There were 2,055 patients in each group after propensity-score matching. The results indicated that the non-glucocorticoid group was not significantly associated with reduced mortality in patients with heart failure during the 14-day follow-up period [HRs = .901, 95% confidence interval (CI) = .767–1.059]. During the follow-up periods of 15–30 and 15–90 days, the mortality risk was significantly lower in the non-glucocorticoid group than in the glucocorticoid group (HRs = .497 and 95% CI = .370–.668, and HRs = .400 and 95% CI = .310–.517, respectively). Subgroup analyses indicated no interaction among each stratification variable and glucocorticoid use.

**Conclusion:** Glucocorticoid use was associated with an increased mortality risk in critically ill patients with heart failure.

## Introduction

Heart failure (HF) is caused by abnormalities in heart structure and function, is characterized by water and sodium retention, often manifests as congestive pulmonary edema or vena cava congestion, and has typical symptoms of dyspnea, edema, and fatigue (Ponikowski et al., 20162016). It has become one of the most common causes of death worldwide, and has gradually become a serious public health problem due to the aging population and its increasing prevalence in the elderly (Vergaro et al., 2019). However, in addition to the aggravation of physiological symptoms, severe HF can also be accompanied by oliguria, electrolyte imbalance, and renal function deterioration. Conventional treatments often fail to significantly relieve symptoms, which increases the difficulty of treatment and the mortality risk.

The relationship between glucocorticoids and the cardiovascular system is well known to be complex. Studies have found that in the treatment of patients with acute decompensated heart failure (ADHF) and diuretic resistance, glucocorticoid use can enhance the effect of diuresis, increase urine output, and improve renal function (Massari et al., 2012). One study assessed a case of a diuretic-resistant patient with ADHF treated using methylprednisolone as a conventional treatment. After 3 days of glucocorticoid treatment the HF symptoms were significantly relieved, and the level of brain natriuretic peptide had decreased by 46% (Massari et al., 2012). A study by Bayliss found that glucocorticoids can promote urinary sodium excretion in patients with congestive HF and induce serum sodium to return to normal levels (Bayliss, 1966). The mechanism of the aforementioned beneficial effects may be related to a 30%–50% increase in renal blood flow and improved renal function (Raisz et al., 1957; Pechet et al., 1959). Studies of HF by Liu et al. found that patients in the glucocorticoid group had an increased urine output and glomerular filtration rate (GFR) compared with those in the placebo group, and these effects were observed after 3 days of glucocorticoid use (Liu et al., 2006a). There are a few studies that have suggested that glucocorticoids can be used safely in the short term and can lead to improvements in the clinical treatment of patients with ADHF (Liu and Liu, 2014). The results of a large, randomized, controlled clinical trial of methylprednisolone for treating acute myocardial infarction indicated that glucocorticoid use reduced the mortality risk in patients with new myocardial infarction by 50% (Stubbs, 1986). However, using glucocorticoids for HF also has certain adverse effects, such as increasing the risk of nosocomial infection, which makes this intervention controversial (Dec, 2007; Mosterd and Hoes, 2007). Glucocorticoids are the drugs most frequently associated with adverse drug events in hospitalized patients in the United States (AHRQ, 2006). Metabolic and cardiovascular adverse events are among the most common and serious adverse events caused by glucocorticoids (Fardet and Fève, 2014).

Research on the association between glucocorticoid use and all-cause mortality in patients with HF in intensive care units has produced unclear results. The aim of this study was to determine the associations of glucocorticoid use with 30- and 90-day all-cause mortality risk in patients with HF.

## Materials and methods

### Data collection

The data analyzed in this study were extracted from the Medical Information Mart for Intensive Care-III (MIMIC-III) database. This large database was established in 2003 by the Beth Israel Deaconess Medical Center and Massachusetts Institute of Technology. The latest version (version 1.4) of the MIMIC-III database collects admission data on more than 40,000 patients during 2001–2012. The database contains detailed patient information, including basic patient demographics, laboratory test results, imaging data, comorbidities, and other medical information (Yang et al., 2020). The database also includes times of admission, discharge, and death. Information on patients who died after discharge can be obtained from the social security database. We completed the relevant courses and passed the examination to obtain a certificate to access the database (certificate number: 45848365).

Patients are fully deidentified in the MIMIC-III database, and so this study did not require approval from the ethics committees of the hospitals. We used Structured Query Language to extract information on variables from the MIMIC-III database. Variables for which the proportion of missing data exceeded 10% were excluded, and missing values for the remaining variables were calculated using the multiple imputation method. The variables in this study included basic patient demographics, laboratory test results, comorbidities, and vital-sign variables (Wu et al., 2021). Demographic information included age and sex. Laboratory test results included potassium, sodium, hemoglobin, lymphocyte, platelet count, activated partial thromboplastin time (APTT), white blood cell (WBC) count, oxygen saturation (SpO2), and glucose. Vital signs included heart rate (HR), systolic blood pressure (SBP), diastolic blood pressure (DBP), respiratory rate (Rr), and body temperature (T). Comorbidities included congestive HF, hypertension, chronic pulmonary disease, diabetes, renal failure, liver disease, peptic ulcer, obesity, and anemia.

### Inclusion and exclusion criteria

Patients diagnosed with HF according to the disease codes of the ninth revision of the International Classification of Diseases were included in the study. Exclusion criteria were as follows: 1) age < 18 years, 2) age > 90 years, and 3) not the first hospitalization.

### Patient outcomes

The outcomes of this study were 30- and 90-day all-cause mortality in patients with HF after admission.

### Statistical analysis

The study population was divided into glucocorticoid and non-glucocorticoid groups. Normally distributed continuous variables are expressed as mean ± standard-deviation values, and non-normally distributed continuous variables are expressed as quartiles. Student’s *t*-test and Kruskal–Wallis test were used to assess the significance of differences between the glucocorticoid and non-glucocorticoid groups. Categorical variables are expressed as frequencies and percentages, and the chi-square test was used to compare differences between groups. Propensity-score matching (PSM) was used to address the imbalance of baseline characteristics between the glucocorticoid and non-glucocorticoid groups. Patients were matched at a 1:1 ratio using estimated propensity scores with a .05 caliper width. The standardized mean difference (SMD) was used to assess whether baseline characteristics were balanced, with SMD < .1 indicating that they were. Multicollinearity among covariates was tested using the variance inflation factor (VIF), with VIF < 5 indicating that there was no multicollinearity.

We counted the number of outcome events at different follow-up periods and compared the differences between the two groups. The Kaplan-Meier (K-M) method was used to estimate the 30- and 90-day survival probabilities of patients in the glucocorticoid and non-glucocorticoid groups, and the log-rank test was used to compare differences in survival probabilities between the two groups. According to the intersection of the survival curves between the glucocorticoid and non-glucocorticoid groups, combined with our clinical experience, we divided the 30-day follow-up period into 1–14 and 15–30 days, and the 90-day period into 1–14 and 15–90 days. We performed landmark analyses for endpoints at different follow-up periods, and differences between the glucocorticoid and non-glucocorticoid groups were compared using the log-rank test.

Cox proportional-hazards regression was used to determine the differences in the risk of death between patients in the glucocorticoid and non-glucocorticoid groups, and results were expressed using hazard ratios (HRs) and 95% confidence intervals (95% CIs). We selected the population of the glucocorticoid group as a reference and established three Cox proportional-hazards regression models: 1) Model I, unadjusted model; 2) Model II, included age, sex, HR, SBP, DBP, Rr, T, and SpO2; and 3) Model III, Model II plus potassium, sodium, hemoglobin, lymphocyte, platelet count, APTT, WBC count, glucose, congestive HF, hypertension, chronic pulmonary disease, diabetes, renal failure, liver disease, peptic ulcer, obesity, and anemia. The performance of each model was evaluated by calculating the C statistic.

We also identified prespecified age, sex, congestive HF, diabetes, hypertension, chronic lung disease, renal failure, liver disease, peptic ulcer, obesity, and anemia. Patients were divided into two groups based on age, with the cutoff of 65 years. In each subgroup, Cox proportional-hazards regression analysis was performed after establishing Model III, and the results were presented using forest plots. We also assessed whether each variable interacted with glucocorticoid exposure to impact all-cause mortality in each follow-up period.

A probability value of *p*-value < .05 was considered significant. We used R software (version 4.2.0) for statistical analyses. The R packages used included gtsummary, tableone, dplyr, remotes, jskm, survival, MatchIt, foreign, forestplot, tidyverse, ISwR, car, lattice, MASS, nnet, mice, and cobalt.

## Results

**FIGURE 1:**
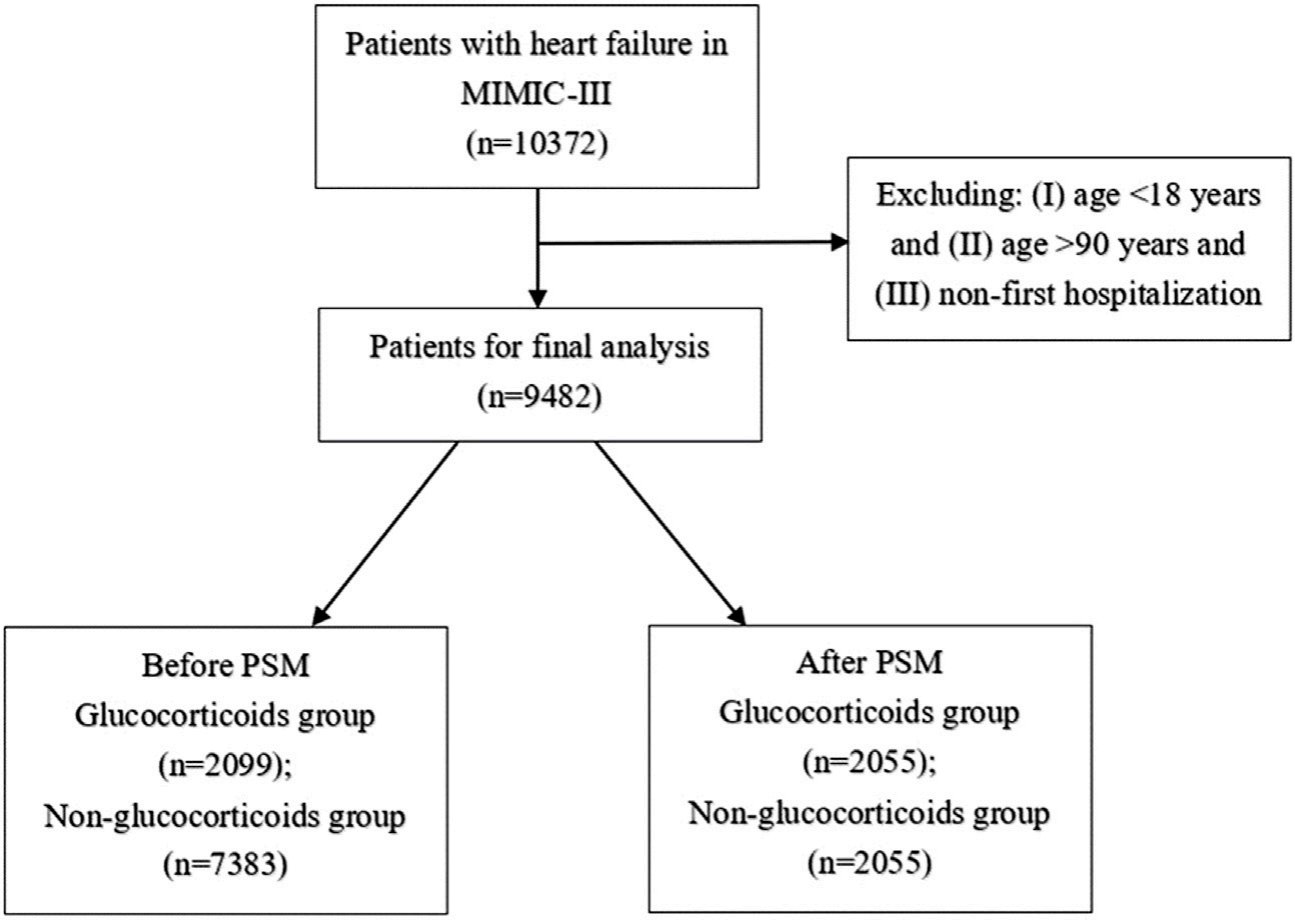
Selection of study population from MIMIC-Ⅲ database.

As shown in Figure 1, 10,372 patients with HF were extracted from the MIMIC-III database. After excluding those aged < 18 and > 90 years and who had previously been hospitalized, 9,482 patients were included in the final analysis. Before PSM, 2,099 patients who had used glucocorticoids were assigned to the glucocorticoid group and 7,383 patients who had not used glucocorticoids were assigned to the non-glucocorticoid group. Table 1 lists the baseline characteristics of the glucocorticoid and non-glucocorticoid groups before matching. Supplementary Table S1 lists the VIFs for each covariate in the three follow-up periods, all of which were < 5, indicating no multicollinearity among the variables. The mean ages of the glucocorticoid and non-glucocorticoid groups were 71.28 and 73.01 years, respectively. The numbers of males and females were 979 and 1,120 in the glucocorticoid group, respectively, and 4,035 and 3,348 in the non-glucocorticoid group. The numbers of patients with congestive HF in the glucocorticoid and non-glucocorticoid groups were 2,048 (97.6%) and 7,066 (95.7%), respectively. The WBC and lymphocyte counts were significantly higher in the glucocorticoid group than in the non-glucocorticoid group. Overall, the baseline characteristics of the two groups of patients were unbalanced for most variables.

**TABLE 1:** Baseline characteristics of population before PSM.

Variables	GCS (*n* = 2099)	Non-GCS (*n* = 7383)	*p*-value	SMD
Potassium (mmol/L)	4.47 ± .79	4.42 ± .71	.011	.061
Sodium (mmol/L)	137.91 ± 4.46	138.15 ± 4.14	.022	.056
Hemoglobin (g/dl)	11.30 ± 1.83	11.50 ± 1.85	<.001	.107
Lymphocyte (%)	12.69 ± 8.58	14.02 ± 7.56	<.001	.165
Platelet count (k/μl)	249.35 ± 112.50	245.18 ± 95.43	.090	.040
APTT (s)	36.06 ± 16.95	38.31 ± 20.67	<.001	.119
WBC, (k/μl)	13.06 ± 20.53	11.76 ± 9.66	<.001	.081
Heart rate (beats/min) (Hr)	87.08 ± 16.34	83.86 ± 15.32	<.001	.203
SBP (mmHg)	116.36 ± 16.48	116.12 ± 16.91	.564	.014
DBP (mmHg)	58.09 ± 10.36	57.24 ± 10.22	.001	.083
Respiration rate (beats/min)	20.30 ± 4.35	19.38 ± 3.95	<.001	.220
Body temperature (°C)	36.72 ± .67	36.76 ± .62	.024	.055
Oxygen saturation, %	96.51 ± 2.84	96.90 ± 2.73	<.001	.142
Glucose, mg/dl	152.10 ± 49.73	140.47 ± 44.04	<.001	.248
Age (years)	71.28 ± 13.62	73.01 ± 13.76	<.001	.127
Gender			<.001	.161
Man	979 (46.6)	4035 (54.7)		
Female	1120 (53.4)	3348 (45.3)		
Congestive heart failure,n (%)			<.001	.104
Yes	2048 (97.6)	7066 (95.7)		
No	51 (2.4)	317 (4.3)		
Hypertension, n (%)			.002	.078
Yes	1215 (57.9)	4556 (61.7)		
No	884 (42.1)	2827 (38.3)		
Chronic pulmonary disease, n (%)			<.001	.356
Yes	960 (45.7)	2127 (28.8)		
No	1139 (54.3)	5256 (71.2)		
Diabetes, n (%)			.028	.055
Yes	715 (34.1)	2710 (36.7)		
No	1384 (65.9)	4673 (63.3)		
Renal failure, n (%)			.325	.025
Yes	571 (27.2)	1927 (26.1)		
No	1528 (72.8)	5456 (73.9)		
Liver disease, n (%)			<.001	.148
Yes	225 (10.7)	486 (6.6)		
No	1874 (89.3)	6897 (93.4)		
Peptic ulcer, n (%)			.908	.006
Yes	17 (.8)	64 (.9)		
No	2082 (99.2)	7319 (99.1)		
Obesity, n (%)			.257	.029
Yes	147 (7.0)	464 (6.3)		
No	1952 (93.0)	6919 (93.7)		
Anemia, n (%)			<.001	.087
Yes	157 (7.5)	395 (5.4)		
No	1942 (92.5)	6988 (94.6)		

**FIGURE 2:**
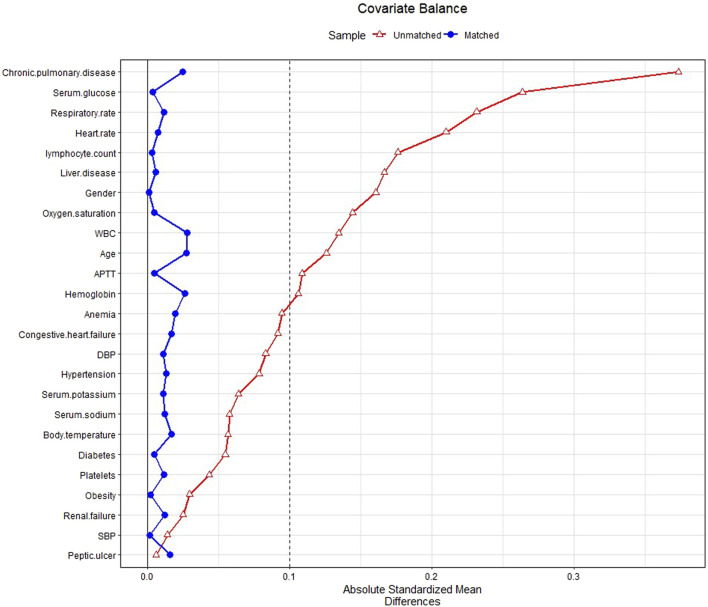
Standardized mean difference (SMD) of variables before and after propensity score matching.

In PSM, 2,055 patients with glucocorticoids were matched with the same number of patients in the non-glucocorticoid group. The baseline characteristics of patients in the glucocorticoid and non-glucocorticoid groups after PSM are listed in Supplementary Table S2. Figure 2 presents the SMD of each covariate before and after PSM. All covariates had SMD < .1 after matching, with *p*-value > .05, indicating that the covariates in the two groups had been balanced.

We assessed differences in all-cause mortality between the glucocorticoid and non-glucocorticoid groups during different follow-up periods. Before PSM, in all follow-up periods (14, 15–30, and 15–90 days), the mortality rate was significantly higher in the glucocorticoid group than in the non-glucocorticoid group (*p*-value < .05). Similarly, the mortality rate was significantly higher in the glucocorticoid group than in the non-glucocorticoid group after PSM; however, there was no significant difference between the two groups during the 14-day follow-up period after PSM (*p*-value > .05). The results are listed in Table 2.

**TABLE 2:** Mortality rates in patients with heart failure in the glucocorticoids group and non-glucocorticoids groups.

	Before PSM			After PSM		
	GCS	Non-GCS	*p*-value	GCS	Non-GCS	*p*-value
Mortality, n (%)						
1–14 day mortality	325 (15.5)	860 (11.6)	.001	314 (15.3)	301 (14.6)	.599
15–30 day mortality	137 (7.7)	192 (2.9)	<.001	133 (7.6)	68 (3.8)	<.001
15–90 day mortality	211 (11.8)	242 (3.7)	<.001	205 (11.7)	85 (4.8)	<.001

**FIGURE 3:**
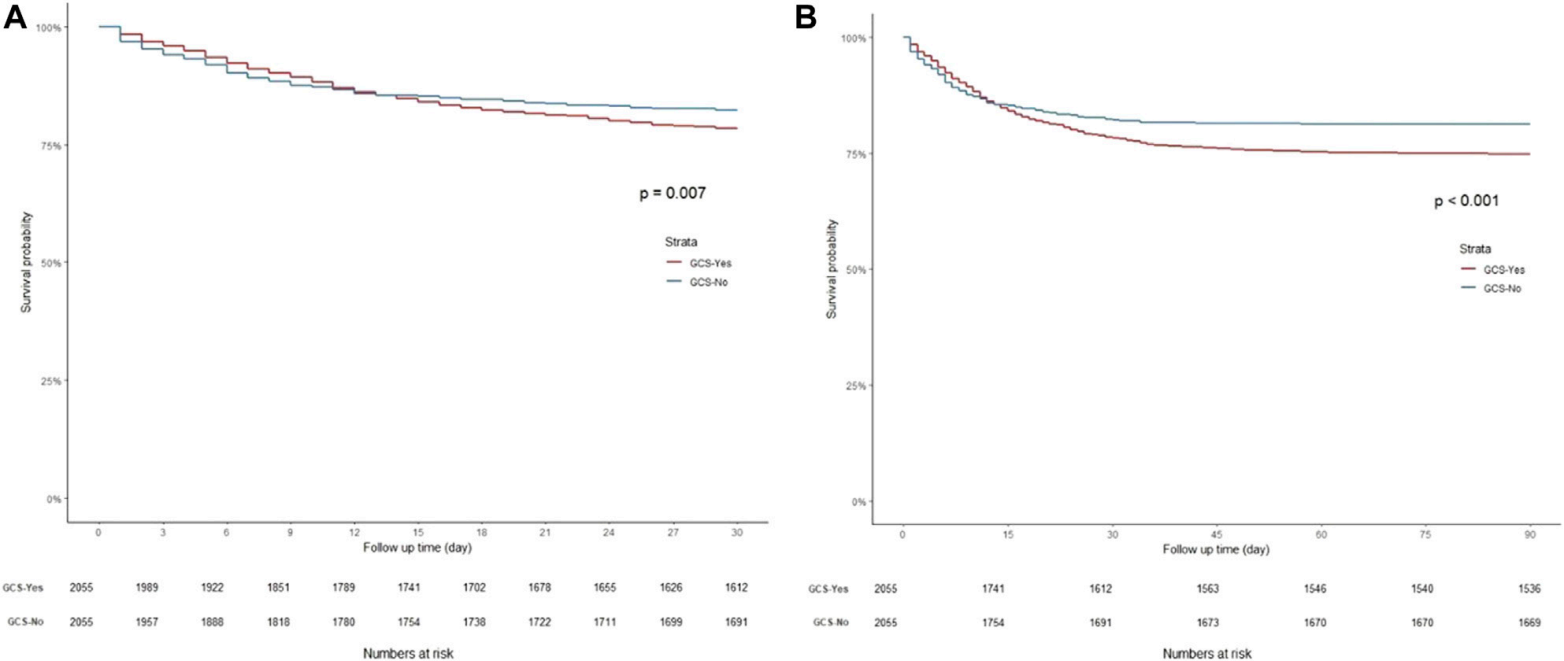
Kaplan-Meier survival curves of non-glucocorticoids group and non-glucocorticoids group after PSM. **(A)** 30-day mortality; **(B)** 90-day mortality.

**FIGURE 4:**
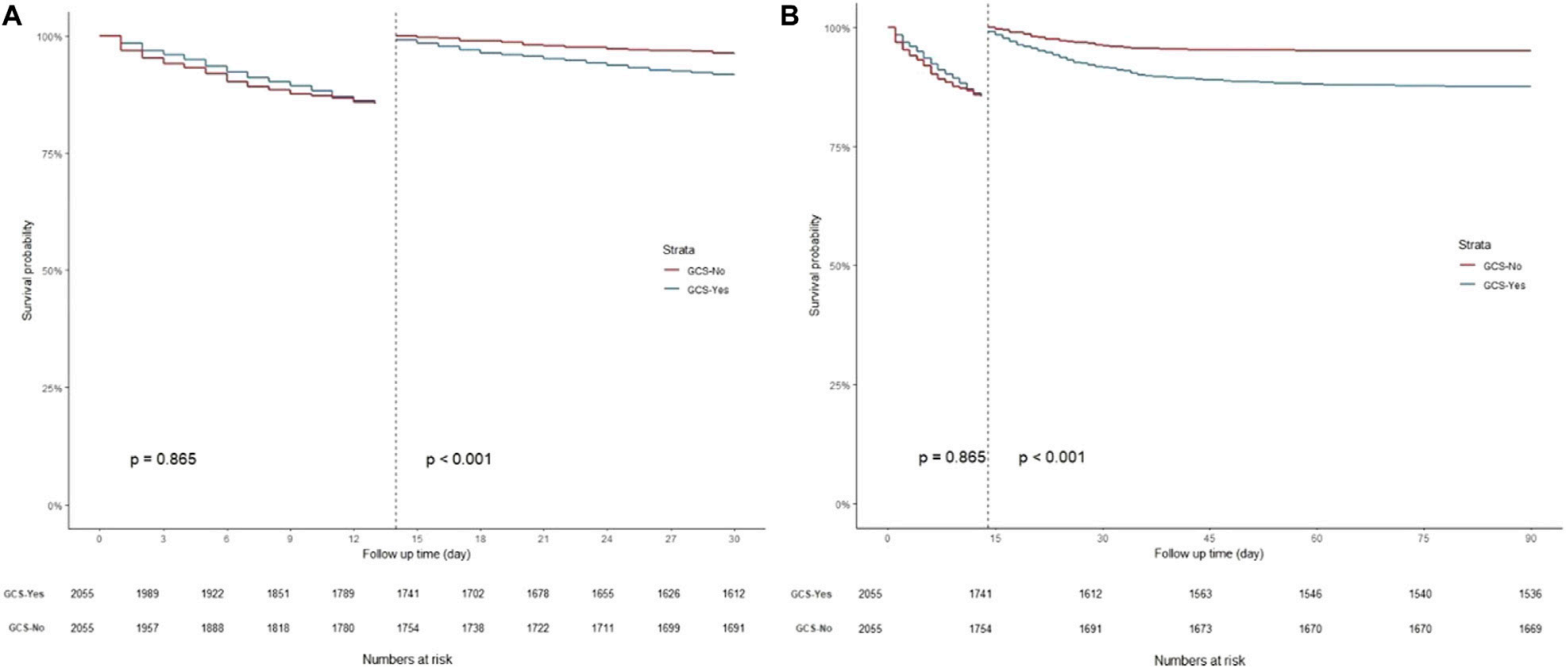
Landmark analysis discriminating between survival probability before and after 14 day of follow-up. **(A)** Landmark analysis during 30-day follow-up; **(B)** Landmark analysis during 90-day follow-up.

K-M curves were used to plot the 30- and 90-day survival probabilities of the glucocorticoid and non-glucocorticoid groups after PSM, which are shown in Figure 3, respectively. Figure 4 present the results of the landmark analysis for 30- and 90-day survival probabilities, respectively. The 30- and 90-day survival probabilities differed significantly between the two patient groups (*p*-value < .05). The survival curves of the glucocorticoid and non-glucocorticoid groups intersected at 14 days. After 14 days, the mortality risk was slightly higher in the non-glucocorticoid group than in the glucocorticoid group, but the difference was not significant (*p*-value > .05). After 15–30 and 15–90 days of follow-up, the mortality risk was significantly lower in the non-glucocorticoid group than in the glucocorticoid group (*p*-value < .05).

In the Cox proportional-hazards regression analysis, we calculated the C statistic for Model III. Before and after PSM, the C statistics for Model III were .739 and .746 for 14 days, respectively, .769 and .760 for 15–30 days, and .775 and .758 for 15–90 day, indicating that the adopted model had good accuracy; the results are listed in Supplementary Table S3. After PSM, within the 14-day follow-up period and with the glucocorticoid group as a reference, the HRs (95% CI) values of the non-glucocorticoid group were .972 (.830–1.139) for Model I, .921 (.785–1.081) for Model II, and .901 (.767–1.059) for Model III. The difference between the two groups for Model III was not significant (*p*-value > .05). During the 15–30 day follow-up period, the risk of death was significantly lower in the non-glucocorticoid group than in the glucocorticoid group (*p*-value < .05); the HRs (95% CI) values of the non-glucocorticoid group were .496 (.370–.665) for Model I, .502 (.374–.672) for Model II, and .497 (.370–.668) for Model III. Similarly, during the 15–90 day follow-up period, the HRs (95% CI) values of the non-glucocorticoid groups were .398 (.309–.513) for Model I, .401 (.311–.517) for Model II, and .400 (.310–.517) for Model III, respectively, and the differences between the risks of death were significant (*p*-value < .05). The results of the Cox proportional-hazards regression analysis are presented in Table 3.

**TABLE 3:** Association between the non-glucocorticoid group and all-cause mortality.

	Before PSM			After PSM		
	GCS	Non-GCS	*p*-value	GCS	Non-GCS	*p*-value
1–14 day mortality		HR (95% CI)				
Events/total n	325/2099	860/7383		314/2055	301/2055	
Model I	References	.747 (.657–.848)	.001	References	.972 (.830–1.139)	.728
Model II	References	.822 (.722–.936)	.003	References	.921 (.785–1.081)	.316
Model III	References	.898 (.786–1.027)	.118	References	.901 (.767–1.059)	.209
15–30 day mortality						
Events/total n	137/1774	192/6523		133/1741	68/1754	
Model I	References	.371 (.298–.462)	<.001	References	.496 (.370–.665)	<.001
Model II	References	.405 (.324–.507)	<.001	References	.502 (.374–.672)	<.001
Model III	References	.472 (.374–.595)	<.001	References	.497 (.370–.668)	<.001
15–90 day mortality						
Events/total n	211/1774	242/6523		205/1741	85/1754	
Model I	References	.300 (.250–.361)	<.001	References	.398 (.309–.513)	<.001
Model II	References	.331 (.274–.400)	<.001	References	.401 (.311–.517)	<.001
Model III	References	.372 (.306–.452)	<.001	References	.400 (.310–.517)	<.001

The subgroup analysis results indicated that stratification variables and glucocorticoid exposure did not interact (P for interaction > .05). Supplementary Figures S1–S3 show the subgroup analysis results. Within 14 days, there were no significant differences in the risk of death between the glucocorticoid and non-glucocorticoid groups for each stratification variable (*p*-value > .05), which was consistent with the overall risk of death, indicating that the results were robust. In the 15–30 and 15–90 day follow-up periods, the risk of death differed significantly between the glucocorticoid and non-glucocorticoid groups (*p*-value < .05) for all stratification variables except for obesity and anemia, which was consistent with the overall risk of death, indicating that the results were robust.

## Discussion

HF is a complex clinical syndrome caused by abnormal changes in cardiac structure and/or function *via* various mechanisms, which results in ventricular systolic and/or diastolic dysfunction. It is a severe manifestation or terminal stage of various heart diseases. In the early stage of HF, the stress response significantly increases the levels of epinephrine, glucagon, and glucocorticoids to above the physiological ranges (Luger et al., 1987). In the later stage, due to low cardiac output, the adrenal cortex is ischemic and hypoxic due to hypoperfusion, so its function of synthesizing corticosteroids is impaired, resulting in decreased blood cortisol.

Glucocorticoids are steroid compounds secreted by the zona fasciae in the adrenal cortex and is mostly regulated by the hypothalamic-pituitary-adrenal axis (Liu et al., 2019a). Physiological doses of glucocorticoids can regulate substance metabolism in the body and maintain its life activities. Supraphysiological doses of glucocorticoids have anti-inflammatory, anti-infective, antishock, and immunosuppressive effects, so they are widely used in clinical practice. The effects of standard treatment drugs are often less strong in the late stage of HF than in the early stage. The adrenal glands are often in an excited state due to repeated acute HF attacks, which eventually leads to a decrease in glucocorticoid secretion, especially in elderly patients with HF accompanied by adrenal insufficiency and infection.

Various animal experiments have found that glucocorticoids can enhance myocardial contractility, which occurs *via* numerous mechanisms. First, the increased release or enhanced effect of endogenous catecholamines indirectly leads to increased myocardial contractility (Tecklenberg et al., 1973; Jain et al., 2004). Second, it may also be caused by β-adrenergic receptor upregulation in the myocardium (Nishimura et al., 1997). Glucocorticoids can also protect kidney function. Previous studies have found that glucocorticoids dilate renal blood vessels, enhance the sensitivity of nephrons to diuretics, and increase the GFR (Liu et al., 2007; Smets et al., 2012; Liu et al., 2016), *via* the following mechanisms: First, glucocorticoids upregulate natriuretic peptide receptor A expression in the inner medullary collecting duct cells to enhance the sensitivity of the kidneys to natriuretic peptides and promote the diuretic effect in patients with HF (Gardner et al., 1986; Damjancic and Vierhapper, 1990a; Liu et al., 2010; Liu et al., 2011). Second, glucocorticoids reduce the production and secretion of vasopressin, and downregulate arginine vasopressin receptor expression (Erkut et al., 1998; Liu et al., 2006b; Greenwood et al., 2015; Zhu et al., 2020); the production of prostaglandins, nitric oxide, and dopamine is also increased, resulting in improved renal blood flow and GFR (Gong et al., 2008; Tokudome et al., 2009; Butts and Phillips, 2013; Liu et al., 2019b). Third, an animal study found that glucocorticoids somewhat affected the synthesis and release of atrial natriuretic peptide (ANP) (Garcia et al., 1985), up-regulating ANP receptors in vascular endothelial cells (Lanier-Smith and Currie, 1990). Likewise, studies have found that glucocorticoids may modulate ANP-mediated natriuresis and diuresis in humans (Damjancic and Vierhapper, 1990b). Glucocorticoids also enhance pulmonary edema clearance. Glucocorticoids can adjust the activity of alveolar epithelial Na+ channel and Na+-K+ ATPase, increase the tension in pulmonary blood vessels, and reduce the permeability of capillaries, so it can increase alveolar fluid clearance and accelerate the curing of pulmonary edema (Barquin et al., 1997; Ingbar et al., 1997).

However, excessive cortisol secretion and the use of various synthetic glucocorticoids increase the incidence rates of diabetes and cardiovascular disease (Plotz et al., 1952). Glucocorticoid use in non-diabetic patients increases their risk of developing diabetes by two-to fourfold (Conn and Poynard, 1994; Gurwitz et al., 1994; Blackburn et al., 2002; Gulliford et al., 2006). The mechanism of glucocorticoid-induced diabetes involves increasing insulin resistance, thereby affecting the glucose metabolism process, which is similar to the mechanism of type 2 diabetes. In the liver, increased insulin resistance can increase basal glucose production. Insulin also promotes intracellular glucose utilization by stimulating glucose transporter type 4, but glucocorticoids interfere with these signaling pathways and with glycogen synthesis. The above two mechanisms may lead to abnormal glucose metabolism and increase blood glucose levels (Ruzzin et al., 2005; Burén et al., 2008). An observational study found that low-density lipoprotein (LDL) was elevated after glucocorticoid therapy in patients with asthma, heart transplant, kidney transplant, and rheumatoid arthritis, which led to abnormal lipid metabolism (Sholter and Armstrong, 2000). An *in vitro* study found that hydrocortisone and dexamethasone alter LDL degradation in fibroblasts and macrophages, which may contribute to atherosclerosis (Henze et al., 1983; Hirsch and Mazzone, 1986).

Dexamethasone is also known to cause cardiotoxicity (de Salvi Guimarães et al., 2017a). Studies have found that long-term dexamethasone use may lead to cardiac fibrosis, increased norepinephrine-induced vasoconstriction, increased apoptosis, and decreased angiogenesis (Qi and Rodrigues, 2007; de Salvi Guimarães et al., 2017a). Dexamethasone can also cause cphy, which is an adaptive ardiac hypertrochange in response to dexamethasone-induced hypertension (Walker, 2007; Stahn and Buttgereit, 2008; Bal et al., 2009). Bézard et al. (2021) assessed the effect of dexamethasone on cardiac function using the Langendorff perfused heart model in healthy rats. The model is free from other confounding factors, allowing for precise studies of how the heart responds to dexamethasone (Sutherland and Hearse, 2000). Their study found that dexamethasone significantly reduced overall left ventricle (LV) function, as well as being caused LV systolic and diastolic dysfunction to various degrees. It is particularly interesting that the above-mentioned changes were also observed in healthy rats (Bézard et al., 2021). Similarly, de Salvi Guimarães et al. (2017b) found that dexamethasone-treated rats also developed multiple circulatory complications, including increased blood pressure, myocardial fibrosis, and cardiomyocyte apoptosis, which led to myocardial remodeling and diastolic dysfunction. That study also confirmed that the mechanisms underlying these changes included impaired calcium handling and activation of the calcineurin signaling pathway. It has also been found that systemic glucocorticoid use may increase the risk of deep-vein thrombosis, especially in patients with pulmonary embolism (Johannesdottir et al., 2013). Despite the effects of glucocorticoids on sodium excretion, diuresis, and increased GFR, glucocorticoid use increased the likelihood of non-acute complications such as diabetes, dyslipidemia, cardiotoxicity, and deep-vein thrombosis, which will be detrimental to the prognosis of patients with HF.

In this study, the WBC count was higher in the glucocorticoid group than in the non-glucocorticoid group (13.06 ± 20.53 vs. 11.76 ± 9.66 k/μl), as was the proportion of patients with chronic respiratory disease (45.7% vs. 28.8%). This suggests that the higher risk of death in the glucocorticoid group is caused by glucocorticoid-use-related diseases, such as chronic respiratory disease and infection. Some studies also found that serum inflammatory markers were associated with the incidence of cardiovascular events, and disease activity was directly related to the occurrence of cardiovascular events (Ross, 1999). We found that the association between glucocorticoid use and increased mortality in patients with HF persisted after adjusting for chronic respiratory disease and WBC counts.

We performed subgroup analyses of age, sex, congestive HF, diabetes, hypertension, chronic lung disease, kidney failure, liver disease, peptic ulcer, obesity, and anemia. The results indicated that there was no interaction between stratification variables and glucocorticoid exposure. The results of each subgroup analysis and the overall results also remained consistent at different follow-up times, indicating that our results were robust.

The highlight of our study was the division of follow-up into two time periods to more precisely account for the occurrence of outcome events. This study had some limitations. First, database limitations meant that we did not perform a subgroup analysis of total glucocorticoid dosage and administration duration. Second, the left ventricle ejection fraction was not analyzed in the study because it had more than 40% missing values. In addition, our study is a single-center study, and we hope that in future studies, multi-center studies can be conducted to obtain more information.

In conclusion, this retrospective study of a large database found that glucocorticoid use was associated with increased all-cause mortality in critically ill patients with HF. Our findings suggest that the use of glucocorticoids should not be recommended for critically ill patients with HF, but this requires further validation in prospective studies or randomized controlled trials.

## Data Availability

Publicly available datasets were analyzed in this study. This data can be found here: https://mimic.mit.edu/.
