# Supplementary material for: Association between glucocorticoid use and all-cause mortality in critically ill patients with heart failure: A cohort study based on the MIMIC-III database
Source: Front Pharmacol. 2023 Jan 12;14:1118551. doi: 10.3389/fphar.2023.1118551 (PMC9877223; doi:10.3389/fphar.2023.1118551)
Supplement: Supplementary file 1 [file DataSheet1.docx]

**Table S1** VIFs for each covariate in the three follow-up periods

| Variables | Variance inflation factor for 14 day mortality | Variance inflation factor for 15-30 day mortality | Variance inflation factor for 15-90 day mortality |
| --- | --- | --- | --- |
| Potassium | 1.119772 | 1.122242 | 1.122242 |
| Sodium | 1.102585 | 1.104482 | 1.104482 |
| Hemoglobin | 1.187123 | 1.181818 | 1.181818 |
| Lymphocyte | 1.058987 | 1.061921 | 1.061921 |
| Platelet count | 1.116024 | 1.120976 | 1.120976 |
| APTT | 1.049828 | 1.042201 | 1.042201 |
| WBC | 1.059874 | 1.063838 | 1.063838 |
| Heart rate | 1.492935 | 1.498835 | 1.498835 |
| SBP | 1.632184 | 1.578229 | 1.578229 |
| DBP | 1.832788 | 1.810559 | 1.810559 |
| Respiration rate | 1.221524 | 1.235647 | 1.235647 |
| Body temperature | 1.187301 | 1.182373 | 1.182373 |
| Oxygen saturation | 1.146787 | 1.136061 | 1.136061 |
| Glucose | 1.148257 | 1.165683 | 1.165683 |
| Age | 1.344995 | 1.362186 | 1.362186 |
| Gender | 1.088179 | 1.092825 | 1.092825 |
| Congestive heart failure | 1.006853 | 1.006066 | 1.006066 |
| Hypertension | 1.163547 | 1.160036 | 1.160036 |
| Chronic pulmonary disease | 1.032819 | 1.045610 | 1.045610 |
| Diabetes | 1.231680 | 1.260958 | 1.260958 |
| Renal failure | 1.187181 | 1.187261 | 1.187261 |
| Liver disease | 1.074325 | 1.076065 | 1.076065 |
| Peptic ulcer | 1.011861 | 1.013437 | 1.013437 |
| Obesity | 1.072127 | 1.078208 | 1.078208 |
| Anemia | 1.025015 | 1.026554 | 1.026554 |

**Table S2** Baseline characteristics of population after PSM

| Variables | GCS (n=2055) | Non-GCS (n=2055) | P-value | SMD |
| --- | --- | --- | --- | --- |
| Potassium (mmol/l) | 4.46 ± 0.78 | 4.46 ± 0.77 | 0.887 | 0.004 |
| Sodium (mmol/l) | 137.92 ± 4.45 | 137.91 ± 4.38 | 0.975 | 0.001 |
| Hemoglobin (g/dl) | 11.33 ± 1.81 | 11.28 ± 1.88 | 0.449 | 0.024 |
| Lymphocyte (%) | 12.77 ± 8.62 | 13.10 ± 7.09 | 0.181 | 0.042 |
| Platelet count (k/uL) | 249.24 ± 111.64 | 250.69 ± 104.93 | 0.670 | 0.013 |
| APTT (s) | 36.12 ± 17.04 | 37.16 ± 18.53 | 0.061 | 0.059 |
| WBC, (k/uL) | 12.50 ± 8.23 | 12.43 ± 9.01 | 0.793 | 0.008 |
| Heart rate (beats/min) (Hr) | 86.92 ± 16.34 | 87.12 ± 16.09 | 0.691 | 0.012 |
| SBP (mmHg) | 116.17 ± 16.33 | 115.82 ± 17.63 | 0.502 | 0.021 |
| DBP (mmHg) | 57.94 ± 10.26 | 57.91 ± 10.57 | 0.936 | 0.002 |
| Respiration rate (beats/min) | 20.25 ± 4.34 | 20.12 ± 4.28 | 0.364 | 0.028 |
| Body temperature (℃) | 36.72 ± 0.67 | 36.73 ± 0.68 | 0.783 | 0.009 |
| Oxygen saturation (%) | 96.54 ± 2.64 | 96.55 ± 3.25 | 0.923 | 0.003 |
| Glucose (mg/dl) | 150.62 ± 47.12 | 149.73 ± 52.96 | 0.566 | 0.018 |
| Age (years) | 71.40 ± 13.54 | 72.00 ± 14.00 | 0.162 | 0.044 |
| Gender |  |  | 1.000 | <0.001 |
| Man | 979 (46.6) | 4035 (54.7) |  |  |
| Female | 1120 (53.4) | 3348 (45.3) |  |  |
| Congestive heart failure ,n (%) |  |  | 1.000 | <0.001 |
| Yes | 2048 (97.6) | 7066 (95.7) |  |  |
| No | 51 (2.4) | 317 (4.3) |  |  |
| Hypertension ,n (%) |  |  | 0.468 | 0.024 |
| Yes | 1215 (57.9) | 4556 (61.7) |  |  |
| No | 884 (42.1) | 2827 (38.3) |  |  |
| Chronic pulmonary disease ,n (%) |  |  | 0.594 | 0.018 |
| Yes | 960 (45.7) | 2127 (28.8) |  |  |
| No | 1139 (54.3) | 5256 (71.2) |  |  |
| Diabetes ,n (%) |  |  | 0.694 | 0.013 |
| Yes | 715 (34.1) | 2710 (36.7) |  |  |
| No | 1384 (65.9) | 4673 (63.3) |  |  |
| Renal failure ,n (%) |  |  | 0.527 | 0.021 |
| Yes | 571 (27.2) | 1927 (26.1) |  |  |
| No | 1528 (72.8) | 5456 (73.9) |  |  |
| Liver disease ,n (%) |  |  | 0.959 | 0.003 |
| Yes | 225 (10.7) | 486 (6.6) |  |  |
| No | 1874 (89.3) | 6897 (93.4) |  |  |
| Peptic ulcer ,n (%) |  |  | 0.625 | 0.020 |
| Yes | 17 (0.8) | 64 (0.9) |  |  |
| No | 2082 (99.2) | 7319 (99.1) |  |  |
| Obesity ,n (%) |  |  | 0.620 | 0.017 |
| Yes | 147 (7.0) | 464 (6.3) |  |  |
| No | 1952 (93.0) | 6919 (93.7) |  |  |
| Anemia ,n (%) |  |  | 0.454 | 0.025 |
| Yes | 157 (7.5) | 395 (5.4) |  |  |
| No | 1942 (92.5) | 6988 (94.6) |  |  |

**Table S3** The C-statistics of Model III

|  | Before PSM | | After PSM | |
| --- | --- | --- | --- | --- |
|  | C-statistic | 95% CI | C-statistic | 95% CI |
| 30-day mortality | 0.743 | 0.729 to 0.757 | 0.734 | 0.716 to 0.751 |
| 90-day mortality | 0.738 | 0.726 to 0.750 | 0.726 | 0.708 to 0.743 |
| 14-day mortality | 0.739 | 0.723 to 0.754 | 0.746 | 0.726 to 0.765 |
| 15-30 day mortality | 0.769 | 0.743 to 0.794 | 0.760 | 0.728 to 0.791 |
| 15-90 day mortality | 0.775 | 0.753 to 0.796 | 0.758 | 0.730 to 0.785 |


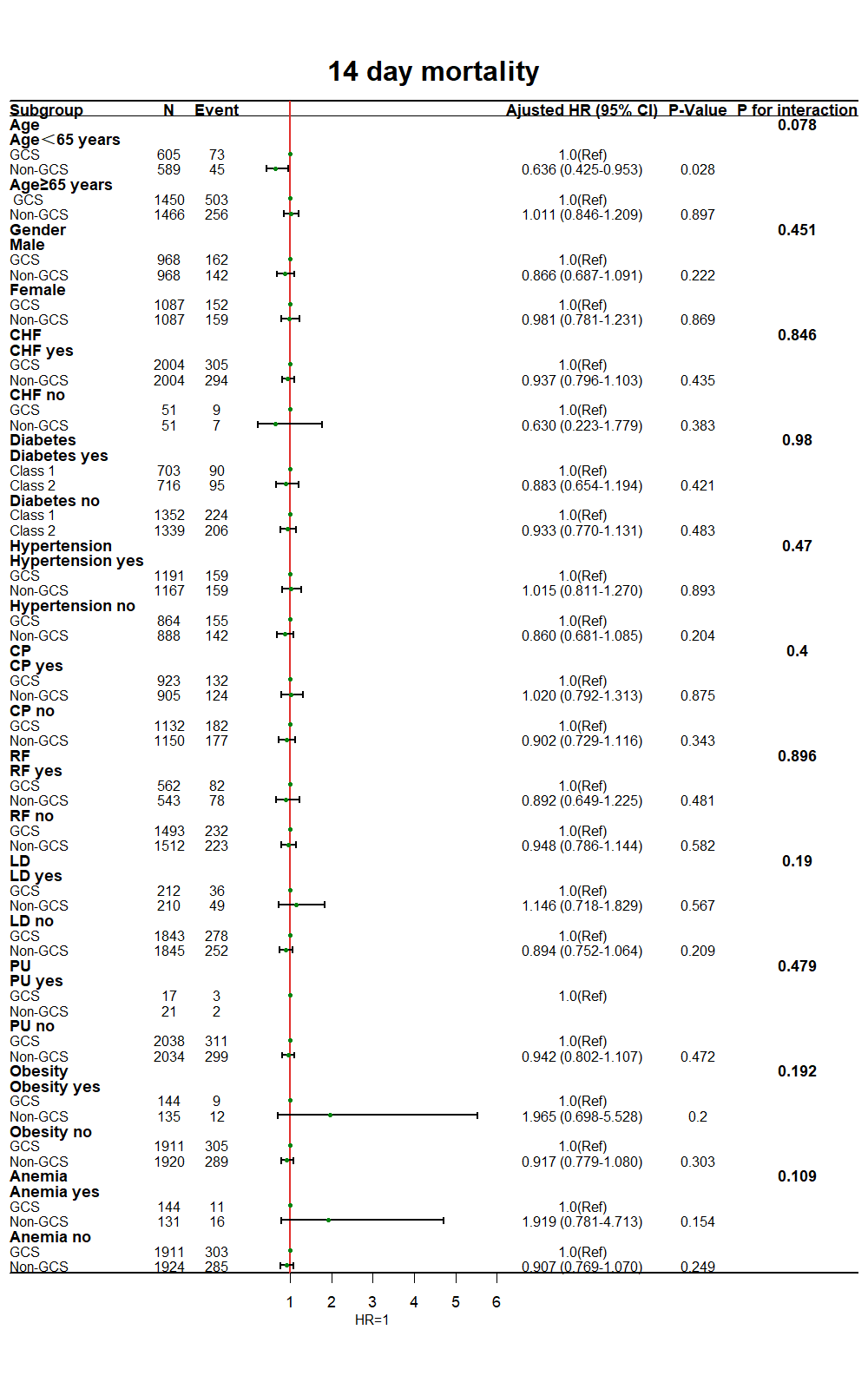


**Figure S1** Subgroup analysis results at 14-day follow-up.


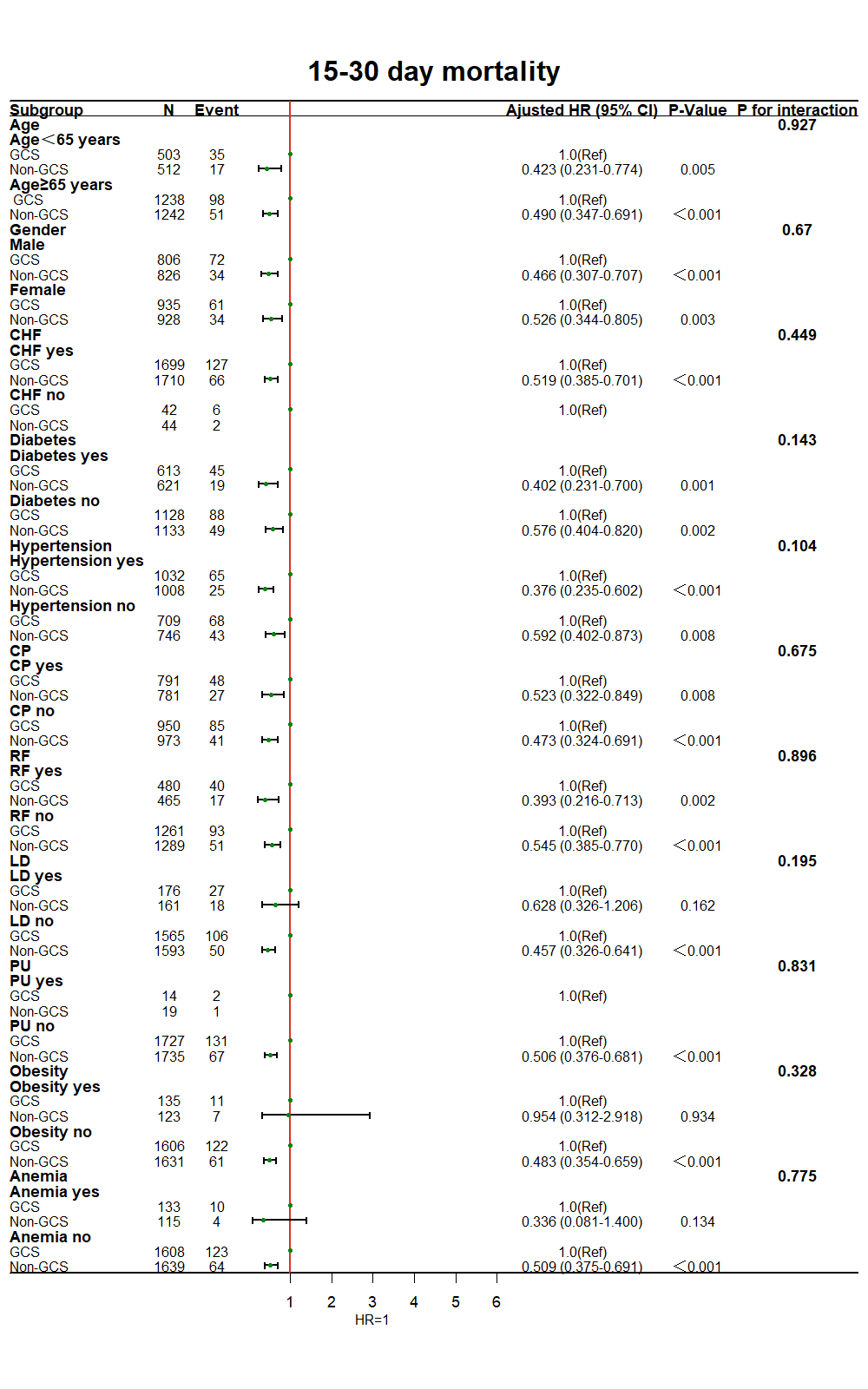


**Figure S2** Subgroup analysis results at 15-30 day follow-up.


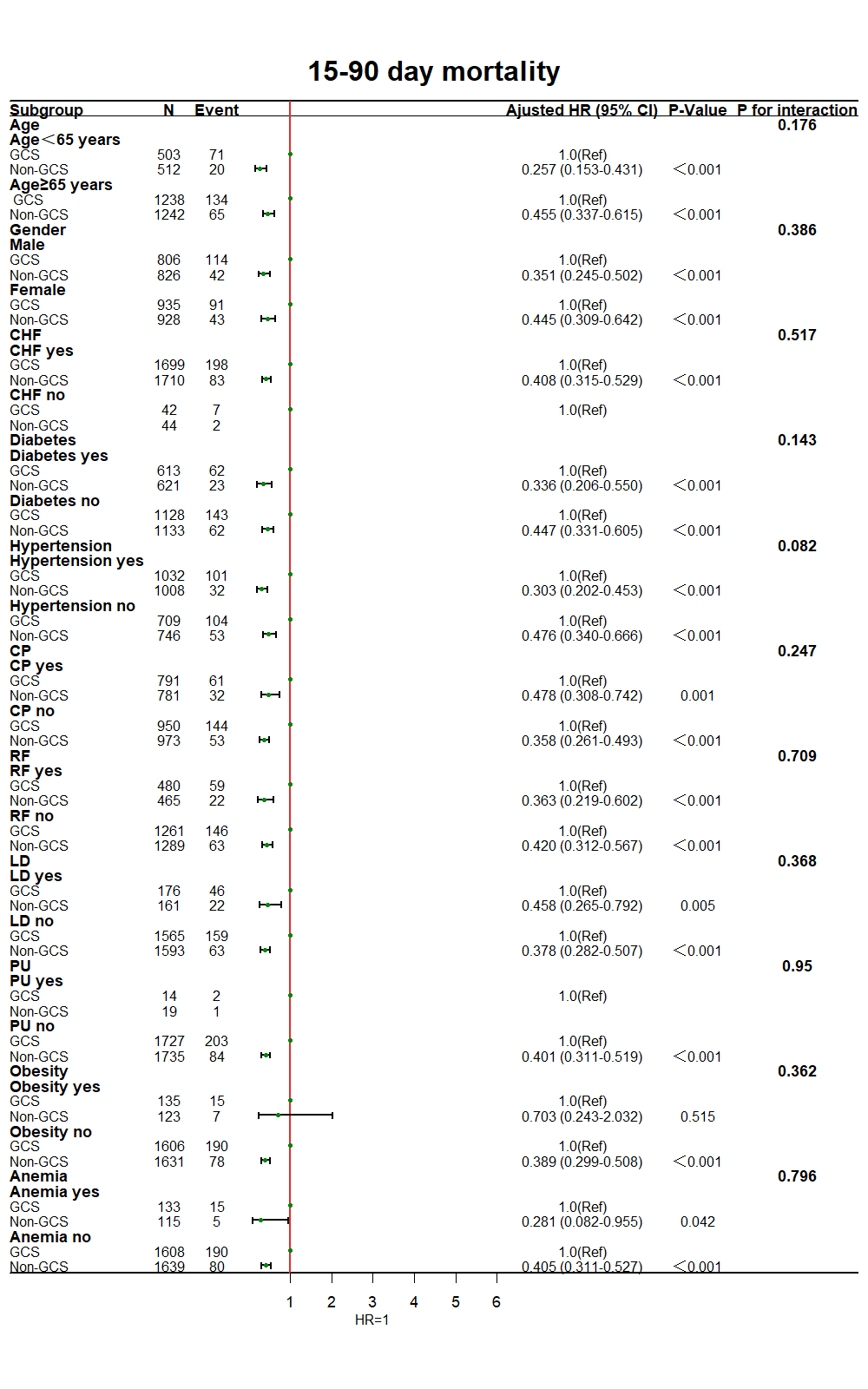


**Figure S3** Subgroup analysis results at 15-90 day follow-up.
